# Erythritol alters phosphotransferase gene expression and inhibits the *in vitro* growth of *Staphylococcus coagulans* isolated from canines with pyoderma

**DOI:** 10.3389/fvets.2023.1272595

**Published:** 2024-01-04

**Authors:** Saki Onishi-Sakamoto, Tadashi Fujii, Keito Watanabe, Reina Makida, Keita Iyori, Yoichi Toyoda, Takumi Tochio, Koji Nishifuji

**Affiliations:** ^1^Cooperative Division of Veterinary Sciences, Graduate School of Agriculture, Tokyo University of Agriculture and Technology, Fuchu, Tokyo, Japan; ^2^Department of Gastroenterology and Hepatology, Fujita Health University, Toyoake, Japan; ^3^Department of Medical Research on Prebiotics and Probiotics, Fujita Health University, Toyoake, Japan; ^4^Cooperative Department of Veterinary Medicine, Faculty of Agriculture, Tokyo University of Agriculture and Technology, Fuchu, Tokyo, Japan; ^5^Research and Development Center, B Food Science Co., Ltd., Chita, Japan; ^6^Dermatological and Laboratory Service for Animals, Vet Derm Tokyo, Fujisawa, Japan; ^7^Division of Animal Life Science, Institute of Agriculture, Tokyo University of Agriculture and Technology, Fuchu, Tokyo, Japan

**Keywords:** erythritol, *Staphylococcus coagulans*, dog, pyoderma, glucose, phosphotransferase system

## Abstract

*Staphylococcus coagulans* (SC) belongs to a group of coagulase-positive staphylococci occasionally isolated from the skin lesions of dogs with pyoderma. We recently revealed that erythritol, a sugar alcohol, inhibited the growth of SC strain JCM7470. This study investigated the molecular mechanisms involved in this growth inhibition of JCM7470 by erythritol, and determine whether erythritol inhibits the growth of SC isolated from the skin of dogs with pyoderma. Comprehensive analysis of the gene expression of JCM7470 in the presence of erythritol revealed that erythritol upregulated the expression of *glcB* and *ptsG* genes, both of which encode phosphotransferase system (PTS) glucoside- and glucose-specific permease C, B, and A domains (EIICBA), respectively, associated with sugar uptake. Moreover, erythritol suppressed *in vitro* growth of all 27 SC strains isolated from the skin lesions of canine pyoderma, including 13 *mecA* gene-positive and 14 *mecA* gene-negative strains. Finally, the growth inhibition of the SC clinical isolates by erythritol was restored by the addition of glucose. In summary, we revealed that erythritol promotes PTS gene expression and suppresses the *in vitro* growth of SC clinical isolates from dogs with pyoderma. Restoration of the erythritol-induced growth inhibition by glucose suggested that glucose starvation may contribute to the growth inhibition of SC.

## Introduction

*Staphylococcus coagulans* (SC), formerly classified as *S. schleiferi* subsp. *coagulans*, is a coagulase-positive staphylococcus primarily isolated from the skin and ear canals of dogs (1–6). Among staphylococci, SC is the second most prevalent cause of skin lesion in dogs with pyoderma after *Staphylococcus pseudintermedius* (SP) (7). In addition to infections in dogs, SC has been reported to cause opportunistic infections in humans (8, 9). Similar to findings in other staphylococcal species (10–13), the emergence of methicillin- and multidrug-resistant SC has been reported (14–17), representing a problem for human and veterinary medicine. Against this background, the development of novel strategies for preventing canine pyoderma using bacteriostatic substrates is anticipated.

Erythritol (1,2,3,4-butanetetrol) is a polyol that is widely used in foods as an artificial sweetener (18). Both erythritol and xylitol, another type of polyol, were shown to inhibit the growth of *Streptococcus mutans, Streptococcus gordonii*, and *Porphyromonas gingivalis*, major human oral commensal bacteria (18–20). Recently, studies have also reported that erythritol inhibited the growth of the human skin commensal bacteria *Corynebacterium minutissimum, Corynebacterium striatum, Staphylococcus epidermidis*, and *Cutibacterium acnes*, as well as the major canine oral commensal bacteria *Porphyromonas gulae* and *Porphyromonas macacae* (21–23).

The mechanisms by which xylitol inhibits *S. mutans* have been well studied (24). *S. mutans* incorporates xylitol via the phosphoenolpyruvate-dependent phosphotransferase system (PEP-PTS) and phosphorylates it to xylitol 5-phosphate. The xylitol 5-phosphate accumulated in the bacteria directly inhibits enzymatic activity related to glycolysis and competes with the phosphor-heat stable protein (24) to indirectly inhibit sugar uptake. Such mechanisms may result in glucose starvation in *S. mutans* and the inhibition of bacterial growth.

Very recently, we revealed that erythritol suppressed *in vitro* growth of the SP and SC strains JCM17571 and JCM7470, respectively (25). Furthermore, erythritol upregulated PEP-PTS-related genes (*ptsG, ppdK*, and *ppdkR*) in SP JCM17571 (26). However, the exact molecular mechanism by which erythritol suppresses the growth of SC has not been elucidated. In this study, we aimed to identify the SC gene clusters whose expression was altered by erythritol. Moreover, we investigated whether erythritol suppresses the growth of SC clinical isolates *in vitro*.

## Materials and methods

### Bacterial strains

SC strain JCM7470 (identical to ATCC 49545) was provided by the Japan Collection of Microorganisms (JCM) and used as a reference strain (2). A previous study revealed that this strain was susceptible to oxacillin and cefoxitin by disk-diffusion tests (27). A total of 27 SC skin isolates from 27 dogs with pyoderma in different private practices and submitted to Vet Derm Tokyo Co., Ltd., for antibiotic susceptibility testing were also used as clinical isolates. The identification of SC was confirmed as follows: The DNA extracted from the 27 bacterial strains was subjected to multiplex PCR for identification of coagulase-positive staphylococcal strains (28). If the band size of the amplicons was identical to that in *S. schleiferi*, the strains were further subjected to a coagulase test using rabbit plasma (Eiken Chemical Co. Ltd., Tokyo, Japan) to identify *S. schleiferi* to the subspecies level (28). The *mecA* gene in the SC clinical isolates was identified by PCR with primer pairs used to identify this gene in *S. aureus*, SP, and *S. schleiferi* isolated from dogs (29). The antimicrobial susceptibility testing was performed by a disk diffusion test using KB Disk™ (Eiken Chemical Co. Ltd.), as described previously (30). The following antimicrobials were used for the susceptibility testing: amoxicillin-clavulanate (AMPC/CVA; 20 or 10 μg/disk), cephalexin (CEX; 30 μg/disk), cefpodoxime (CPDX; 10 μg/disk), enrofloxacin (ERFX; 5 μg/disk), gentamicin (GM; 10 μg/disk), sulfamethoxazole-trimethoprim (ST; 23.75–1.25 μg/disk), clindamycin (CLDM; 2 μg/disk), doxycycline (DOXY; 30 μg/disk), minocycline (MINO; 30 μg/disk), chloramphenicol (CP; 30 μg/disk), and fosfomycin (FOM; 50 μg/disk). Supplementary Table 1 lists the PCR primers used in this study, and Supplementary Table 2 shows the results of the disk diffusion susceptibility tests.

### Bacterial culture

The following experiments were performed in accordance with the methodology used in a previous study (26). A single colony of JCM7470 was inoculated into 3 ml of National Institute of Technology and Evaluation Biological Resource Center (NRBC) #802 medium containing 1% hipolypepton (Fujifilm Wako, Osaka, Japan), 0.2% yeast extract (Nacalai Tesque Inc., Kyoto, Japan), and 0.1% MgSO_4_·7H_2_O (Fujifilm Wako, pH 7.0), and incubated with rotation at 210 rpm until the optical density at 600 nm (OD_600_) reached 3.4. The bacterial suspensions were further diluted 100-fold in NRBC #802 medium with or without 5% (w/w) erythritol (B Food Sciences Co. Ltd., Tokyo, Japan) and incubated at 30°C until OD_600_ reached 0.8–1.0. These experiments were performed in triplicate. We chose an erythritol concentration of 5% in this study as we had observed that erythritol at higher concentrations significantly inhibited the growth of JCM7470 in a previous study (25).

### RNA sequencing (RNA-seq)

RNeasy Mini Kit (Qiagen, Venlo, Netherlands) was used to extract total RNA from the bacterial samples. The total RNA samples were submitted to Bioengineering Lab (Sagamihara, Japan). After removal of ribosomal RNA using riboPOOLS (siTOOLs Biotech, Planegg, Germany), a cDNA library for RNA-seq analysis was generated using MGIEasy RNA Directional Library Prep Set (MGI Tech, Shenzhen, China). The cDNA library was used to construct a circular DNA library using the MGIEasy Circularization Kit (MGI Tech). The cDNA library anchored by DNA Nanoball (DNA) was subjected to sequencing analysis using DNBSEC-G400 (MGI Tech). Nucleic Acid SeQuence Analysis Resource (NASQAR; https://nasqar.abudhabi.nyu.edu) was used for creating principal component analysis (PCA) plots of the triplicate samples and a heatmap to visualize the RNA-seq results. A volcano plot was created using ggVolcanoR (https://ggvolcanor.erc.monash.edu). Protein ANNotation with Z-scoRE (PANNZER2; http://ekhidna2.biocenter.helsinki.fi/sanspanz) was used for gene ontology (GO) analysis to predict the genes up- and downregulated in response to erythritol.

### Reverse-transcription quantitative polymerase chain reaction (RT-qPCR)

Transcriptor First Strand cDNA Synthesis Kit (Roche Diagnostics, Rotkreuz, Switzerland) with random primers was used to synthesize complementary DNA from total RNA extracted from JCM7470 with or without erythritol. Supplementary Table 1 lists the primers used in this study. The primer sets and TB Green^®^ Fast qPCR Mix (Takara Bio, Kusatsu, Japan) were used for RT-qPCR on a Thermal Cycler Dice^®^ Real-Time System III (Takara Bio) with 45 cycles of 95°C for 5 s and 55°C for 60 s. The *recA* gene, which has been validated as an appropriate reference gene for qPCR in SP (31), was used as a reference to evaluate the relative gene expression levels of the other genes. The RNA-seq performed in this study revealed that the *recA* gene expression in SC was consistent regardless of the presence of erythritol (log_2_FC = −0.19, *p* = 0.111), suggesting the validity of the *recA* gene as a reference gene for qPCR in SC. This experiment was performed in triplicate, and mean values were compared among the groups.

### *In vitro* turbidity assay of SC clinical isolates

The SC clinical isolates were pre-cultured in Luria-Bertani (LB) medium (Kanto Chemical Co., Inc., Tokyo, Japan) and diluted fivefold in this medium. Then, 30 μL of the diluted bacterial suspension was mixed with 900 μL of NRBC #802 medium with erythritol at concentrations of 0%, 5%, 10%, and 15% [w/w] in 96-well U-bottomed microplates (Watson Corporation, Tokyo, Japan). The OD_600_ was measured over time using EpochTM2 (Agilent Technologies, Inc., Santa Clara, CA, USA) from 0 to 6 h.

The effect of glucose on the erythritol-induced growth inhibition of SC clinical isolates was analyzed as follows. The SC isolates were incubated in NRBC #802 medium for 2 h and diluted fivefold in NRBC #802 medium. Then, 30 μL of the diluted bacterial suspension was mixed with 900 μL of NRBC #802 medium containing 0% erythritol and 0% glucose, 0% erythritol and 0.1% glucose, 10% erythritol and 0.1% glucose, 10% erythritol and 1% glucose, or 10% erythritol and 0% glucose, and 200 μL was inoculated into each 96-well plate. The OD_600_ was measured over time for up to 6 h. The experiments analyzing growth were performed in triplicate, and mean values were compared among the groups.

### Statistical analysis

Empirical Analysis of Digital Gene Expression Data in R (edgeR) exactTest was used to compare gene expression levels analyzed by RNA-seq. GraphPad Prism 9 software (GraphPad Software Inc., San Diego, CA, USA) was used for the following statistical analysis. Welch's t-test was used to compare transcription levels of *glcB* and *ptsG* genes between the groups, and the effect of erythritol on *mecA* gene-positive and -negative SC strains. Dunnett's test was used to compare the turbidity between SC strains incubated in the presence or absence of erythritol and/or glucose. A *p*-value of less than 0.05 was considered statistically significant.

## Results

### Comprehensive gene expression analysis of SC JCM7470 in response to erythritol

We first performed RNA-seq analysis to investigate the molecular mechanism behind the growth inhibition of JCM7470 by erythritol. After filtering the raw sequencing reads, we obtained 16,589,804, 16,962,572, and 27,123,064 clean reads of the transcriptome in control samples. By contrast, there were 17,294,425, 18,110,786, and 15,713,381 clean reads in the erythritol-treated samples.

**Figure 1:**
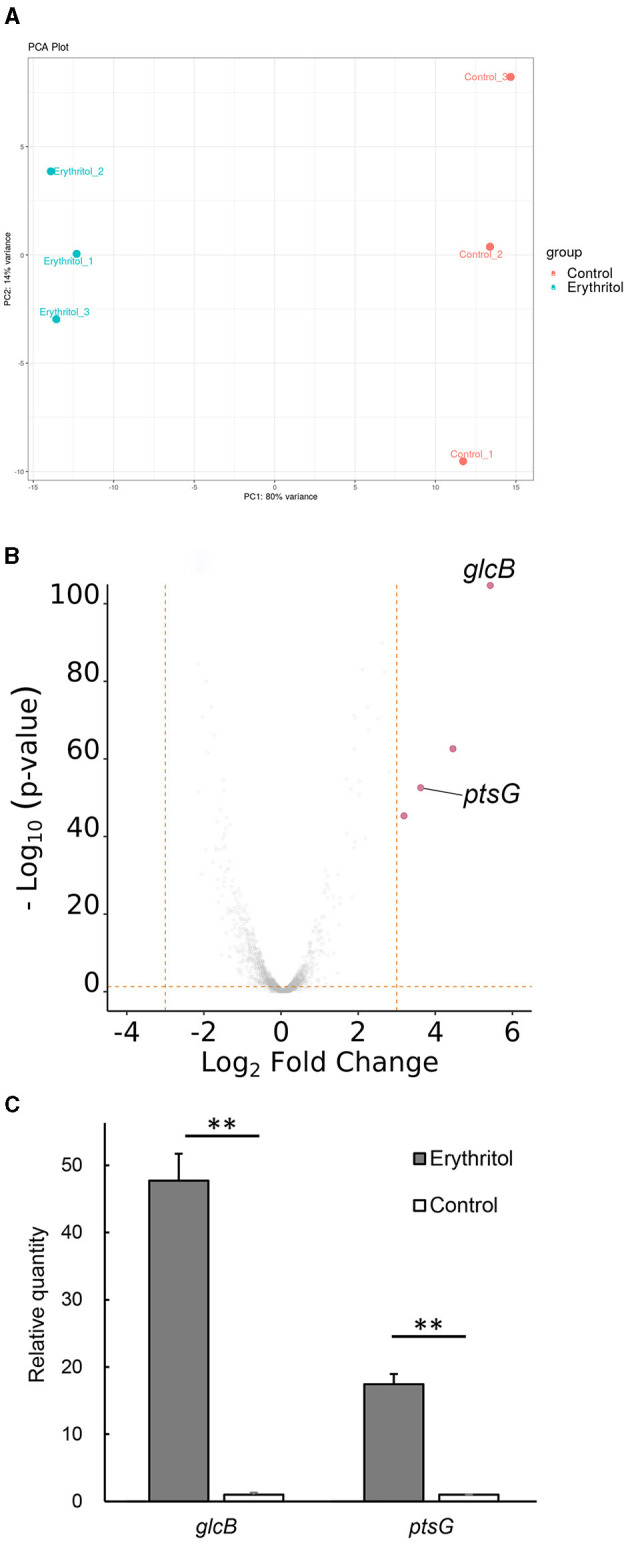
Gene expression analysis. **(A)** PCA plot shows a clear split between the control and erythritol-treated *Staphylococcus coagulans* JCM7470, with 80% of the variance explained by PC1. **(B)** The volcano plot shows differences in expression levels of up- and downregulated genes in JCM7470 treated with erythritol. The genes upregulated >10-fold (log_2_FC > 3.322, *p* < 0.05) in response to erythritol are highlighted in red. **(C)** Comparison of the expression levels of *glcB* and *ptsG* genes in JCM7470 treated with erythritol or control medium, as determined by RT-qPCR. ^**^p < 0.01.

The calculated gene expression levels [|log2 fold change (log_2_FC)| > 1, *p* < 0.05] identified a total of 162 differentially expressed genes, including 60 upregulated and 102 downregulated genes, in JCM7470 following erythritol treatment. The PCA plot with 80% of the variance explained by PC1 exhibited a clear split between the control and erythritol-treated samples (Figure 1A). The heatmap is shown in Supplementary Figure 1.

### Erythritol upregulated the expression of glucose-specific phosphotransferase system genes in JCM7470

Among the 162 genes in JCM7470 differentially expressed in response to erythritol, only four were upregulated more than 10-fold (log_2_FC > 3.322) in the presence of erythritol. Conversely, no genes were downregulated more than 10-fold in the presence of erythritol.

Supplementary Table 3 shows the predicted functional descriptions and GO biological processes for the top 15 up- and downregulated genes in response to erythritol. The GO analysis revealed that two of the four most upregulated genes were *glcB* [log_2_FC = 5.459, *p* < 0.001, positive predictive value (PPV) = 0.71] and *ptsG* (log_2_FC = 3.727, *p* < 0.001, PPV = 0.71), both of which encode PTS transporter subunit IIBC (Figure 1B). Meanwhile, the other two most upregulated genes encode hypothetical proteins for which the associated biological processes are unknown (Figure 1B). RT-qPCR revealed that transcription levels of *glcB* and *ptsG* genes in the erythritol-treated group were significantly higher than those in the control group (*glcB, p* = 0.0024; *ptsG, p* = 0.0028, Figure 1C). The log_2_ fold changes of *glcB* and *ptsG* in the erythritol-treated group relative to the levels in the control group were 5.57 ± 0.06 and 4.12 ± 0.06 (mean ± SE), respectively.

### Glucose supplementation restored erythritol-induced growth inhibition of SC clinical isolates

**Figure 2:**
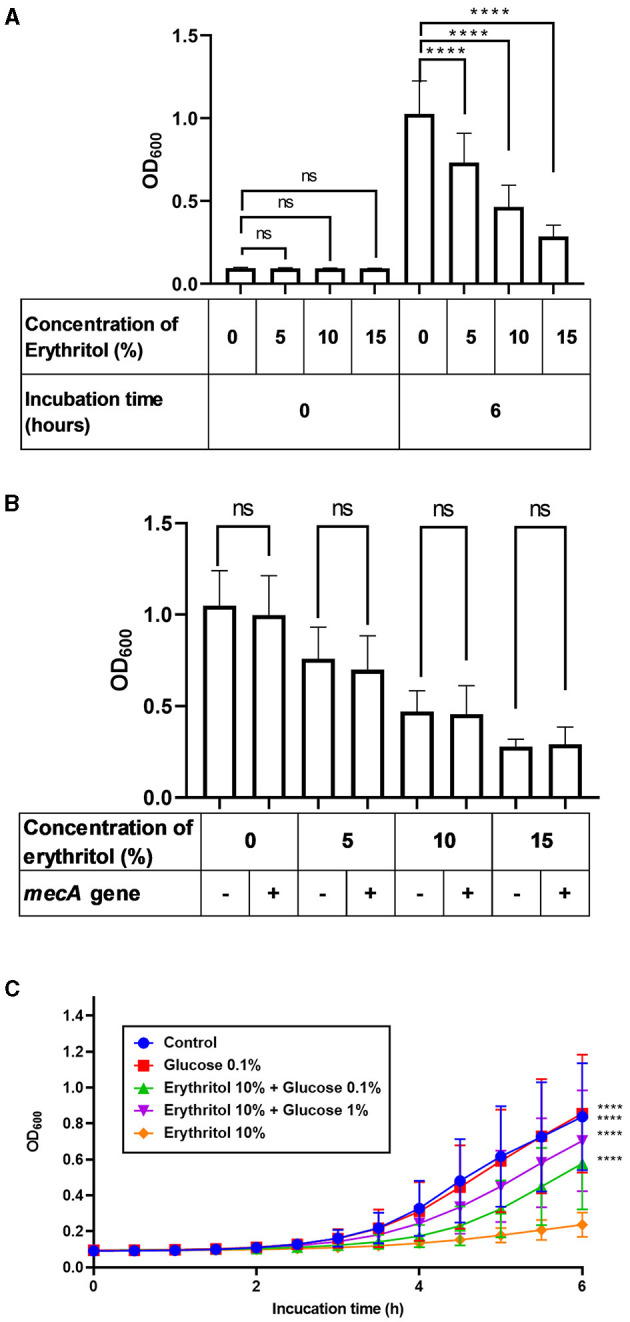
Restoration of erythritol-induced growth inhibition of SC clinical isolates by glucose supplementation. **(A)** Bacterial turbidity of the SC clinical isolates (*n* = 27) immediately or 6 h after incubation with different doses of erythritol. **(B)** Comparison of bacterial turbidity between *mecA* gene-positive and -negative SC strains 6 h after incubation with different doses of erythritol. **(C)** Fluctuation of the bacterial turbidity of the SC strains incubated with different doses of erythritol and glucose. ns, not significant; ****p < 0.0001.

We next investigated whether erythritol suppresses the growth of SC isolated from clinical lesions of canine pyoderma. *In vitro* turbidity assay revealed that the turbidity increased over time up to 6 h in both the control group and the erythritol-supplemented groups, regardless of whether the strains carried the *mecA* gene (Supplementary Figure 2). The turbidity of 27 SC clinical isolates incubated for 6 h in the presence of 5% (0.728 ± 0.172, *p* < 0.0001), 10% (0.460 ± 0.130, *p* < 0.0001), and 15% erythritol (0.283 ± 0.070, *p* < 0.0001) was significantly lower than the level upon incubation in the absence of erythritol (1.023 ± 0.193). Moreover, erythritol suppressed the growth of the SC clinical isolates in a concentration-dependent manner (*p* < 0.0001). In contrast, there were no significant differences in the baseline bacterial turbidity in the SC groups between erythritol-supplemented groups and the control group (*p* > 0.05) (Figure 2A). Furthermore, there were no significant differences in turbidity between the *mecA* gene-positive strains (*n* = 13) and *mecA* gene-negative strains (*n* = 14) supplemented with 0% (*p* = 0.295), 5% (*p* = 0.332), 10% (*p* = 0.703), and 15% erythritol (*p* = 0.709) (Figure 2B).

Considering the upregulation of PTS-related genes and growth inhibition, we wondered whether carbohydrate starvation in response to erythritol caused the growth inhibition of the SC clinical isolates. Therefore, we investigated whether glucose supplementation of the culture media of the SC clinical isolates would overcome the growth inhibition induced by erythritol. We found that the turbidity of the SC clinical isolates supplemented with 10% erythritol and 0.1% (0.577 ± 0.250, *p* < 0.0001) or 1% glucose (0.704 ± 0.275, *p* < 0.0001) was significantly higher than that upon supplementation with 10% erythritol alone (Figure 2C).

## Discussion

### Erythritol may induce glucose starvation in SC

The present study revealed that erythritol significantly upregulated the expression of *glcB* and *ptsG* encoding PTS transporter subunit IIBC in the SC strain. GO analysis predicted that the transcripts of these two genes function as glycoside- and glucose-specific enzyme II components EIICBA, respectively. The upregulation of *ptsG* gene expression in response to erythritol in SC was in agreement with the data obtained in our recent study using an SP strain (26). The EIICBA are membrane permeases that play significant roles in the uptake of carbohydrates into the bacterial cytoplasm (32, 33). EIIA, EIIB, and EIIC usually specifically incorporate one substrate or closely related carbohydrates into bacterial cytoplasm (34).

Furthermore, restoration of the erythritol-induced growth suppression by glucose supplementation implies that glucose starvation in response to erythritol may cause the upregulation of PTS-related gene expression and result in the growth inhibition of SC. It was reported that, in *S. mutans*, xylitol 5-phosphate, a metabolite derived from xylitol, directly inhibits glycolytic enzymes and competes with glucose 6-phosphate, a glucose metabolite incorporated into glycolysis (24). We speculate that erythritol or its metabolites also compete for the glycolytic enzyme in SC, although the exact erythritol uptake and metabolic pathways in the staphylococci have yet to be determined.

Previous studies revealed that the *ptsG* operon of *S. carnosus* consists of two adjacent genes, *glcA* and *glcB*, which encode IICBA^Glc^1 and IICBA^Glc^2, respectively (32, 33, 35). Analysis of the deduced amino acid sequence suggested that the *ptsG* gene in SC (NCBI WP_ 050331035.1) consists of two components, PTS-II-BC-glcB (glucose-specific IIBC component) and PTS-EIIA-1, while the *glcB* gene in SC (NCBI WP_ 0503356536.1) consists of two components, PTS-II-BC-glcB and NagE (IIA component). Efforts should be made to ensure consistency in the gene nomenclature between *S. carnosus* and SC. Nevertheless, the sequence analysis suggested that these two genes encode enzyme II components crucial in carbohydrate uptake into SC.

### Differences in erythritol-induced alteration of gene expression profiles in SC and SP

In the SP strain, erythritol upregulated *ppdK* and *ppdkR*, which are other PTS-related genes predicted to encode pyruvate phosphate dikinase (PPDK) and PPDK regulatory protein (PPDKR), respectively (26). The same study also revealed that erythritol downregulated the expression of *pur* operon genes involved in the synthesis of inosinic acid (IMP) leading to purine biosynthesis in the SP strain. However, such changes were not recognized in the present study using the SC strains. Possible reasons for this discrepancy include differences in the bacterial species or that such changes are a late phenomenon occurring in response to glucose starvation. Indeed, PPDK and PPDKR contribute to the regeneration of PEP necessary to reactivate PTS (36, 37), and phosphoribosyl pyrophosphate, the precursor of IMP, is a metabolite derived from glucose 6-phosphate through the pentose phosphate pathway (38). The expression of genes encoding *vraTSR*, which are associated with resistance to β-lactams and glycopeptides in *S. aureus* (39–43), and *sgtB*, which is involved in proteoglycan biosynthesis in *S. aureus* (44), was also upregulated in the SP strain. The present study revealed slight increases in *vraS, sgtB*, and *vraR* gene expression (|log_2_FC| < 1.9) (Supplementary Table 3). However, the biological significance of these changes induced by erythritol in SC was not identified in this study because the changes in the susceptibility of SC to antibiotics were not evaluated.

### Future perspectives on the application of erythritol clinically

We also revealed that erythritol inhibited the growth of SC isolated from the skin lesions of canines with pyoderma. A previous study revealed that erythritol has a bacteriostatic effect on bacteria associated with canine periodontal disease (45). Based on these findings, we assumed that SC strains whose growth was inhibited in the presence of erythritol may grow on agar plates for colony counting and thus did not analyze the effect of erythritol on colony-forming units. Studies have reported that erythritol was more efficient at inhibiting the growth of human oral commensal streptococci than xylitol (18), while such efficacy of erythritol against SP and SC clinical isolates was similar to that of xylitol (25).

Unlike in humans, the safe dosage range of xylitol in dogs is narrow and there is a risk of xylitol toxicity such as hypoglycemia and acute liver failure, which raises safety concerns (46, 47). In contrast, erythritol was less likely to cause hypoglycemia and appeared to be safe for dogs, as determined by oral toxicity studies (48, 49). Therefore, topical application of erythritol is expected to have the potential to alleviate the clinical severity of canine pyoderma caused by SC or to prevent its recurrence. Future clinical trials with topically applied erythritol for canine pyoderma are expected. It is also anticipated that erythritol can prevent SC infections via contaminated medical equipment in humans and animals.

## Conclusion

Our findings suggest that glucose starvation in response to erythritol contributes to growth inhibition in SC. Our findings also suggest the potential of erythritol in preventing SC-associated cutaneous infections in dogs and the contamination of medical equipment.

## Data availability statement

The RNA-seq data are available at Gene Expression Omnibus (GEO) in The National Center for Biotechnology Information with GEO accession number GSE245057. All other data are available from the corresponding author upon reasonable request.

## Author contributions

SO-S: Formal analysis, Investigation, Writing – original draft. TF: Formal analysis, Conceptualization, Methodology, Writing – review & editing. KW: Writing – review & editing, Investigation. RM: Investigation, Writing – review & editing. KI: Investigation, Writing – review & editing, Formal analysis. YT: Writing – review & editing, Methodology. TT: Writing – review & editing, Conceptualization. KN: Conceptualization, Writing – review & editing.
