# Supplementary material for: Erythritol alters phosphotransferase gene expression and inhibits the in vitro growth of Staphylococcus coagulans isolated from canines with pyoderma
Source: Front Vet Sci. 2024 Jan 4;10:1272595. doi: 10.3389/fvets.2023.1272595 (PMC10794667; doi:10.3389/fvets.2023.1272595)
Supplement: Supplementary file 1 [file Data_Sheet_1.docx]

**Erythritol alters phosphotransferase gene expression and inhibits the *in vitro* growth of *Staphylococcus coagulans* isolated from canines** **with pyoderma**

Saki Onishi-Sakamoto^1^, Tadashi Fujii (ORCID: 000-0001-7466-895X)^2,3^, Keito Watanabe^4^, Reina Makita^5^, Keita Iyori (ORCID: 0000-0003-1977-4201)^6^, Yoichi Toyoda^6^, Takumi Tochio (ORCID: 0000-0003-3206-4116)^2,3^, Koji Nishifuji (ORCID: 0000-0003-4583-5485)^7,*^

**Supplemental Figure Legends**

Suppl. Fig. 1 Heatmap showing differentially expressed genes in *S. coagulans* JCM7470 incubated with erythritol or control.

Suppl. Fig. 2 Time-course fluctuation of bacterial turbidity of all SC strains, *mecA* gene-positive strains, and *mecA* gene-negative strains incubated with different doses of erythritol.
