# Supplementary material for: Erythritol alters phosphotransferase gene expression and inhibits the in vitro growth of Staphylococcus coagulans isolated from canines with pyoderma
Source: Front Vet Sci. 2024 Jan 4;10:1272595. doi: 10.3389/fvets.2023.1272595 (PMC10794667; doi:10.3389/fvets.2023.1272595)
Supplement: Supplementary file 2 [file Table_1.DOCX]

**Supplemental Table 1** Primers used for multiplex PCR and RT-qPCR

| Genes | Primer set | Sequence (5′–3′) | PCR product length (bp) | Reference |
| --- | --- | --- | --- | --- |
| *S. schleiferi* | sch-F | AATGGCTACAATGATAATCACTAA | 526 | Sasaki et al., 2010 |
|  | sch-R | CATATCTGTCTTTCGGCGCG |  |  |
| *mecA* | mecA-F | TAGTCCGTAACCTGAATCAGC | 131 | Gonzalez-Domínguez et al., 2020 |
|  | mecA-R | TGCTATCCACCCTCAAACAG |  |  |
| *glcB* | glcB-F | CGGCAATAACATGCAAGCGA | 173 | This study |
|  | glcB-R | CAGGGGCAACAACTGAGACA |  |  |
| *ptsG* | ptsG-F | AATCCGTGAAGGTGCGAACT | 160 | This study |
|  | ptsG-R | TGTTAAAGCAGCAGAGCCCA |  |  |
| *recA* | recA-F | CATTAGGGGTTGGCGGCTAT | 154 | This study |
|  | recA-R | GGATCCAACGCATGTTCAGC |  |  |

**Supplemental Table 2** Results of disk diffusion susceptibility tests of 27 SC clinical isolates

| **Antimicrobial agent** | **Number of susceptible strains (%)** | **Number of intermediate strains (%)** | **Number of resistant strains (%)** |
| --- | --- | --- | --- |
| Amoxycillin-clavulanate (AMPC/CVA) | 14 (51.9) | 0 (0.0) | 13 (48.1) |
| Cefalexin (CEX) | 14 (51.9) | 0 (0.0) | 13 (48.1) |
| Cefpodoxime (CPDX) | 14 (51.9) | 0 (0.0) | 13 (48.1) |
| Cefovecin (CFV) | 14 (51.9) | 0 (0.0) | 13 (48.1) |
| Faropenem (FRPM) | 14 (51.9) | 0 (0.0) | 13 (48.1) |
| Enrofloxacin (ERFX) | 8 (29.6) | 4 (14.8) | 15 55.6) |
| Marbofloxacin (MBFX) | 8 829.69 | 1 (3.7) | 18 (66.7) |
| Gentamicin (GM) | 26 (96.2) | 1 (3.7) | 0 (0.0) |
| Sulfamethoxazole-trimethoprim (ST) | 26 (96.2) | 0 (0.0) | 1 (3.7) |
| Clindamycin (CLDM) | 27 (100.0) | 0 (0.0) | 0 (0.0) |
| Lincomycin (LCM) | 24 (88.9) | 3 (11.1) | 0 (0.0) |
| Erythromycin (EM) | 26 (96.2) | 1 (3.7) | 0 (0.0) |
| Doxycycline (DOXY) | 25 (92.6) | 1 (3.7) | 1 (3.7) |
| Minomycin (MINO) | 26 (96.2) | 1 (3.7) | 0 (0.0) |
| Chloramphenicol (CP) | 27 (100.0) | 0 (0.0) | 0 (0.0) |
| Fosfomycin (FOM) | 25 (92.6) | 0 (0.0) | 2 (7.4) |
| Rifampicin (RFP) | 27 (100.0) | 0 (0.0) | 0 (0.0) |
| Mupirocin (MUP) | 27 (100.0) | 0 (0.0) | 0 (0.0) |

**Supplemental Table 3** Top 15 upregulated genes (top table) and top 15 downregulated genes (bottom table) by erythritol

| **Log_2_FC** | **p-value** | **Gene_id** | **Gene product** | **Gene　name** | **Log_2_FC** | | **p-value** | | |
| --- | --- | --- | --- | --- | --- | --- | --- | --- | --- |
|  |  |  |  |  | **Estimated PPV (>0.3)** | **Description** | **Estimated PPV (>0.5)** | **GO-id** | **Description** |
| 5.458723 | 0 | RS07705 | WP_103356536.1 glucose-specific PTS transporter subunit IIBC [*Staphylococcus coagulans*] | *glcB* | 0.71 | PTS system glucoside-specific EIICBA component | 0.82 | GO:1904659 | Glucose transmembrane transport |
|  |  |  |  |  |  |  | 0.71 | GO:0009401 | Phosphoenolpyruvate-dependent sugar phosphotransferase system |
|  |  |  |  |  |  |  | 0.59 | GO:0016310 | Phosphorylation |
| 4.458835 | 2.38E-63 | RS04520 | WP_050329956.1 MULTISPECIES: hypothetical protein [*Staphylococcus*] |  |  |  |  |  |  |
| 3.72684 | 1.1256E-203 | RS02480 | WP_050331035.1 MULTISPECIES: glucose-specific PTS transporter subunit IIBC [*Staphylococcus*] | *ptsG* | 0.71 | PTS system glucose-specific EIICBA component | 0.82 | GO:1904659 | Glucose transmembrane transport |
|  |  |  |  |  |  |  | 0.72 | GO:0009401 | Phosphoenolpyruvate-dependent sugar phosphotransferase system |
|  |  |  |  |  |  |  | 0.59 | GO:0016310 | Phosphorylation |
| 3.622495 | 2.72011E-53 | RS10000 | WP_103356650.1 hypothetical protein [*Staphylococcus coagulans*] |  |  |  |  |  |  |
| 3.189249 | 4.64706E-46 | RS10595 | WP_050345836.1 MULTISPECIES: DUF5011 domain-containing protein [*Staphylococcus*] |  | 0.51 | Chitinase |  |  |  |
| 2.809425 | 3.33277E-57 | RS05230 | WP_050331696.1 MULTISPECIES: M50 family metallopeptidase [*Staphylococcus*] |  | 0.56 | M50 family metallopeptidase |  |  |  |
| 2.685329 | 3.35118E-83 | RS06505 | WP_060830091.1 MULTISPECIES: ABC transporter permease [*Staphylococcus*] | *potC* | 0.36 | Polyamine transporter subunit membrane component of ABC superfamily | 0.8 | GO:0015675 | Nickel cation transport |
|  |  |  |  |  |  |  | 0.55 | GO:0055085 | Transmembrane transport |
| 2.627947 | 1.33573E-90 | RS07855 | WP_060829246.1 MULTISPECIES: 6-phospho-beta-glucosidase [*Staphylococcus*] | *bglA* | 0.41 | 6-Phospho-beta-glucosidase | 0.59 | GO:0005975 | Carbohydrate metabolic process |
| 2.5193 | 4.53056E-71 | RS00320 | WP_050345538.1 MULTISPECIES: DUF4930 family protein [*Staphylococcus*] |  |  |  |  |  |  |
| 2.272453 | 4.88885E-68 | RS06510 | WP_060830092.1 MULTISPECIES: ABC transporter permease [*Staphylococcus*] | *potB* | 0.37 | Spermidine/putrescine ABC transporter, permease protein PotB | 0.8 | GO:0015675 | Nickel cation transport |
|  |  |  |  |  |  |  | 0.55 | GO:0055085 | Transmembrane transport |
| 2.244224 | 4.38419E-74 | RS06500 | WP_103356422.1 spermidine/putrescine ABC transporter substrate-binding protein [*Staphylococcus coagulans*] | *potD* | 0.39 | ABC transporter, periplasmic spermidine putrescine-binding protein potD | 0.77 | GO:0015846 | Polyamine transport |
| 2.194719 | 2.64047E-40 | RS07105 | WP_103356465.1 hypothetical protein [*Staphylococcus coagulans*] |  |  |  |  |  |  |
| 2.11264 | 8.99701E-84 | RS06580 | WP_103356427.1 cytochrome d ubiquinol oxidase subunit II [*Staphylococcus coagulans*] | *cydB* | 0.56 | Cytochrome d ubiquinol oxidase subunit II |  |  |  |
| 2.036686 | 6.61248E-55 | RS00580 | WP_050330548.1 MULTISPECIES: cell wall-active antibiotic response protein LiaF [*Staphylococcus*] | *liaF* | 0.78 | Transporter associated with VraSR |  |  |  |
| 2.024772 | 1.43702E-51 | RS00575 | WP_103356076.1 hypothetical protein [*Staphylococcus coagulans*] |  |  |  |  |  |  |

| **Log_2_FC** | **p-value** | **Gene_id** | **Gene product** | **Gene name** | **Log_2_FC** | | **p-value** | | |
| --- | --- | --- | --- | --- | --- | --- | --- | --- | --- |
|  |  |  |  |  | **Estimated PPV (>0.3)** | **Description** | **Estimated PPV (>0.5)** | **GO-id** | **Description** |
| -2.161983 | 4.53102E-85 | RS05670 | WP_050331847.1 MULTISPECIES: histidine racemase CntK [*Staphylococcus*] | *cntK* | 0.88 | Histidine racemase CntK |  |  |  |
| -2.134603 | 2.0437E-52 | RS07265 | WP_103356475.1 dihydroxy-acid dehydratase [*Staphylococcus coagulans*] | *ilvD* | 0.64 | Dihydroxy-acid dehydratase | 0.73 | GO:0009099 | Valine biosynthetic process |
|  |  |  |  |  |  |  | 0.73 | GO:0009097 | Isoleucine biosynthetic process |
| -2.058082 | 5.31739E-31 | RS07255 | WP_103356473.1 acetolactate synthase small subunit [*Staphylococcus coagulans*] | *ilvN* | 0.58 | Acetolactate synthase | 0.71 | GO:0009082 | Branched-chain amino acid biosynthetic process |
|  |  |  |  |  |  |  | 0.67 | GO:0050790 | Regulation of catalytic activity |
| -2.030628 | 1.53E-71 | RS05665 | WP_060830428.1 MULTISPECIES: staphylopine biosynthesis enzyme CntL [*Staphylococcus*] | *cntL* | 1.00 | Staphylopine biosynthesis enzyme CntL | 0.8 | GO:0003941 | L-Serine ammonia-lyase activity |
|  |  |  |  |  |  |  | 0.66 | GO:0051539 | 4 iron, 4 sulfur cluster binding |
|  |  |  |  |  |  |  | 0.54 | GO:0046872 | Metal ion binding |
| -1.950413 | 2.2481E-37 | RS04710 | WP_103356336.1 ABC transporter substrate-binding protein [*Staphylococcus coagulans*] | *appA* | 0.39 | ABC transporter substrate-binding protein | 0.55 | GO:0055085 | Transmembrane transport |
| -1.934143 | 9.68059E-81 | RS05660 | WP_060830427.1 MULTISPECIES: staphylopine biosynthesis dehydrogenase [*Staphylococcus*] | *cntM* | 1.00 | Staphylopine biosynthesis dehydrogenase |  |  |  |
| -1.91262 | 2.18462E-62 | RS03395 | WP_060829327.1 MULTISPECIES: malonate decarboxylase subunit alpha [*Staphylococcus*] |  | 0.75 | Propionate CoA-transferase | 0.83 | GO:0046952 | Ketone body catabolic process |
| -1.80658 | 3.63221E-74 | RS05655 | WP_060830426.1 MULTISPECIES: nickel ABC transporter substrate-binding protein [*Staphylococcus*] | *cntA* | 0.82 | Staphylopine-dependent metal ABC transporter substrate-binding protein CntA | 0.8 | GO:0015675 | Nickel cation transport |
|  |  |  |  |  |  |  | 0.55 | GO:0055085 | Transmembrane transport |
| -1.769642 | 3.97638E-34 | RS07260 | WP_103356474.1 biosynthetic-type acetolactate synthase large subunit [*Staphylococcus coagulans*] | *ilvB* | 0.50 | Acetolactate synthase | 0.73 | GO:0009234 | Menaquinone biosynthetic process |
|  |  |  |  |  |  |  | 0.73 | GO:0009099 | Valine biosynthetic process |
|  |  |  |  |  |  |  | 0.71 | GO:0009097 | Isoleucine biosynthetic process |
| -1.723089 | 7.05474E-67 | RS11500 | WP_103356732.1 B domain-containing protein, partial [*Staphylococcus coagulans*] |  | 0.75 | Immunoglobulin G binding protein A |  |  |  |
| -1.686865 | 6.23818E-38 | RS01610 | WP_060829378.1 MULTISPECIES: class I SAM-dependent methyltransferase [*Staphylococcus*] |  | 0.3 | Class I SAM-dependent methyltransferase | 0.63 | GO:0032259 | Methylation |
| -1.677735 | 2.87713E-37 | RS04725 | WP_050345215.1 MULTISPECIES: ABC transporter permease [*Staphylococcus*] |  | 0.38 | Oligopeptide transport system permease | 0.8 | GO:0015675 | Nickel cation transport |
|  |  |  |  |  |  |  | 0.55 | GO:0055085 | Transmembrane transport |
| -1.65569 | 1.54645E-44 | RS03415 | WP_103356256.1 ABC transporter ATP-binding protein [*Staphylococcus coagulans*] | *ssuB* | 0.36 | Sulfonate/nitrate/taurine transport system ATP-binding protein |  |  |  |
| -1.632906 | 1.37193E-29 | RS07250 | WP_103356472.1 ketol-acid reductoisomerase [*Staphylococcus coagulans*] | *ilvC* | 0.67 | Ketol-acid reductoisomerase (NADP(+)) | 0.73 | GO:0009099 | Valine biosynthetic process |
|  |  |  |  |  |  |  | 0.72 | GO:0009097 | Isoleucine biosynthetic process |
| -1.623715 | 8.65638E-24 | RS04720 | WP_050345214.1 MULTISPECIES: ATP-binding cassette domain-containing protein [*Staphylococcus*] | oppF | 0.33 | Oligopeptide transport ATP-binding protein OppF | 0.71 | GO:0015833 | Peptide transport |
