# Supplementary figures and images for: Erythritol alters phosphotransferase gene expression and inhibits the in vitro growth of Staphylococcus coagulans isolated from canines with pyoderma
Source: Front Vet Sci. 2024 Jan 4;10:1272595. doi: 10.3389/fvets.2023.1272595 (PMC10794667; doi:10.3389/fvets.2023.1272595)

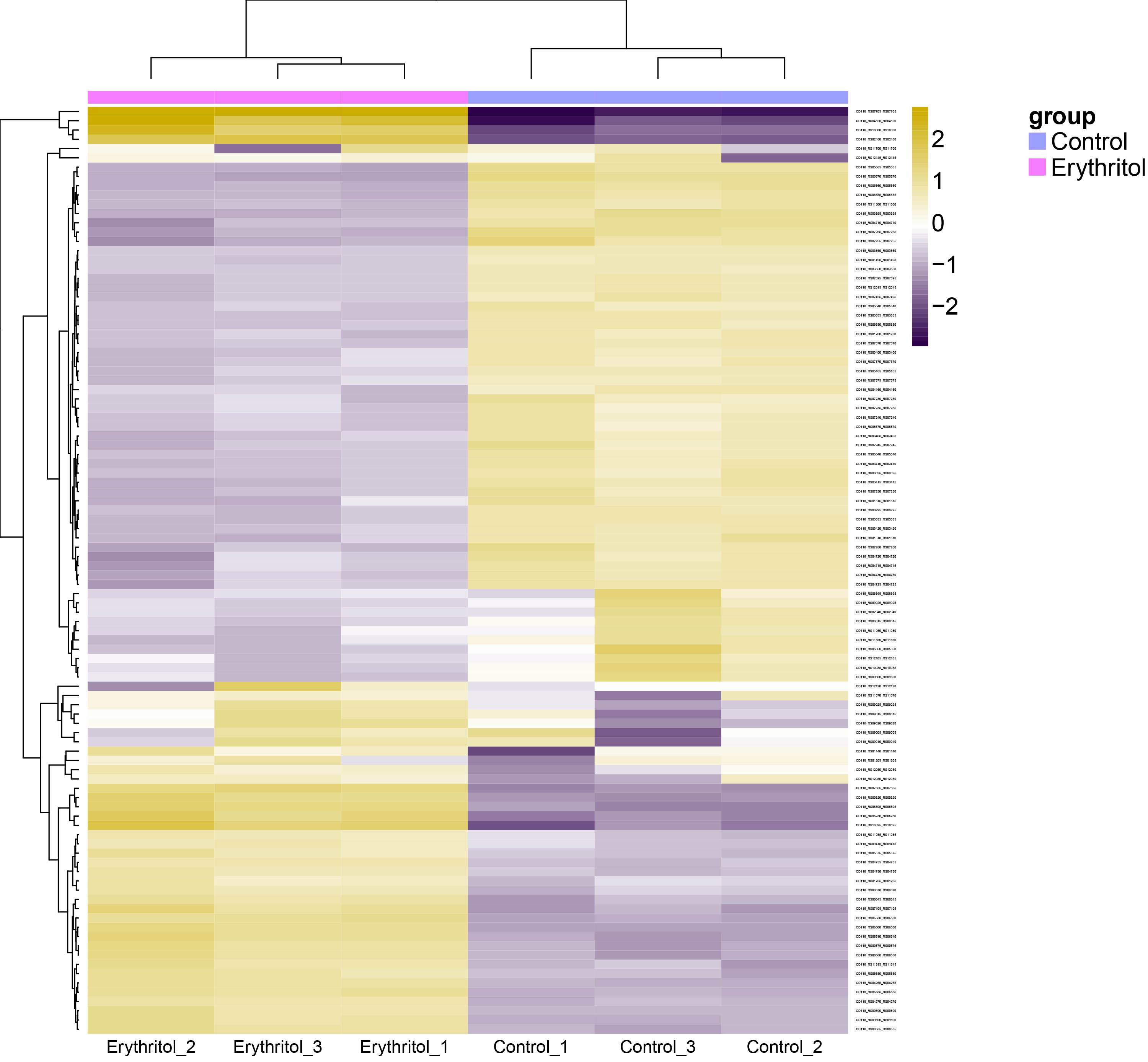

Supplement: Supplementary file 3 [file Image_1.JPEG]

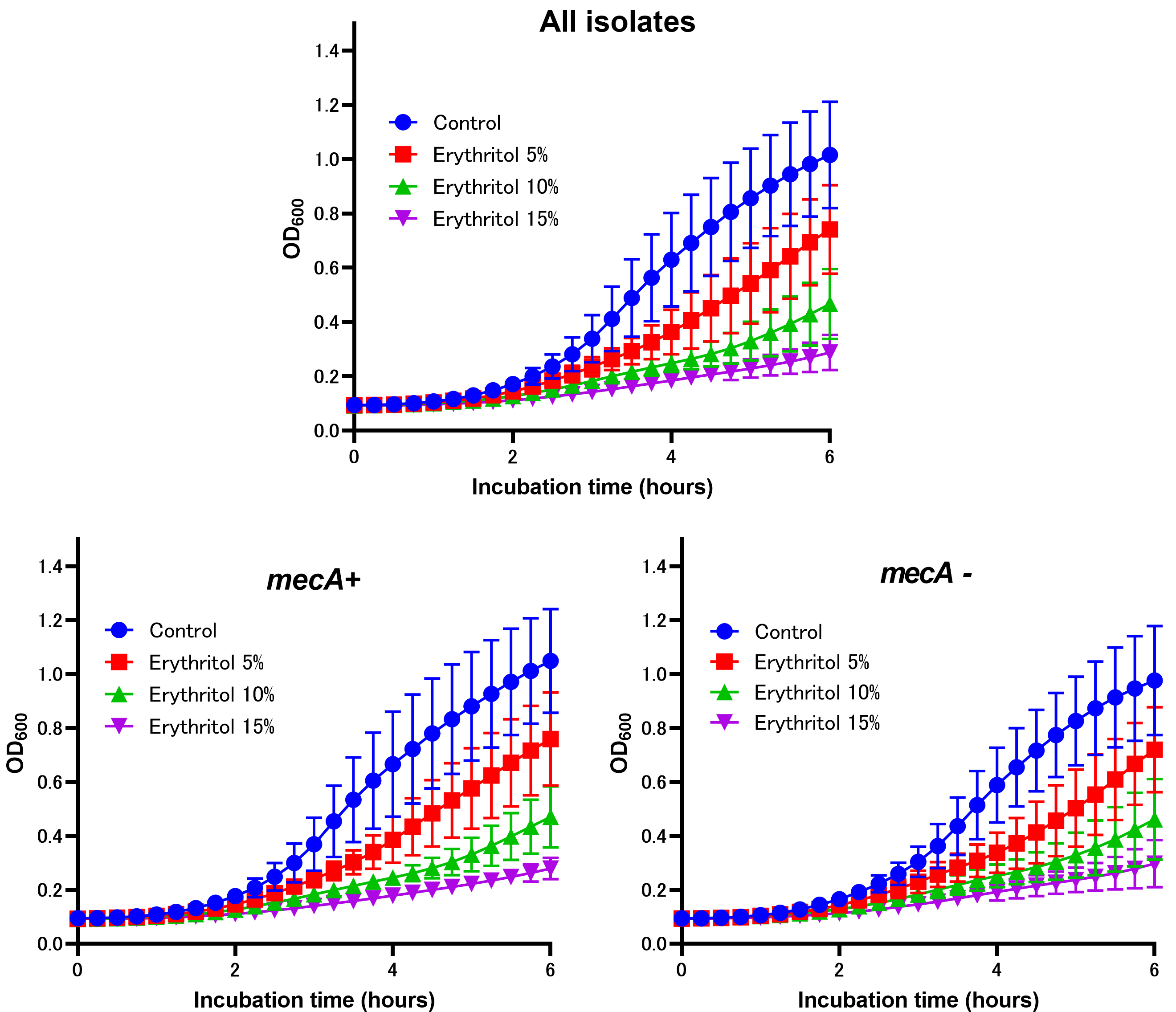

Supplement: Supplementary file 4 [file Image_2.JPEG]
